# The Effect of Training in Primary Health Care Centers on Medical Students' Clinical Skills

**DOI:** 10.5402/2013/403181

**Published:** 2013-04-18

**Authors:** Faisal Abdullatif Alnasir, Ahmed Abdel-Karim Jaradat

**Affiliations:** Department of Family & Community Medicine, College of Medicine and Medical Sciences, Arabian Gulf University, P.O. Box 26671, Manama, Bahrain

## Abstract

Medical students' effective clinical skills training are an important goal of any medical school. When adequate, graduate doctors will have sufficient skills to consult a patient by taking proper history and conducting appropriate physical examination. The question under scrutiny is the optimal place for providing such training. Since the aim is to graduate general physicians, many literatures highlighted the importance of implementing such training in the primary health care centers. A special clinical skills training program was developed for the Year 4 pre-clerkship medical students of the Arabian Gulf University during the academic year 2011-2012. It was important for these students to acquire certain skills before transferring to the clerkship phase where they deal directly with patients. For the 130 students involved in this study, a self-assessment and clinical exam were conducted at the beginning and end of the program. The study showed that students benefited greatly from this training program with significant differences between their preexisting known skills and clinical skills acquired by the end of the program. Primary care centers are ideal places for optimal training because of small group training setting that is one tutor to two students and of the advantage that students face real patient environment.

## 1. Background

The College of Medicine and Medical Science (CMMS) of the Arabian Gulf University (AGU) is a community-oriented medical school. Since its establishment in 1983, it has graduated more than 22 batches of doctors. The main objective of the school is to produce generalist physicians who are well aware of patients' medical, social, and psychological problems. Much feedback from external examiners on several occasions indicates that students in the clerkship phase lack clinical skills despite inclusion of such, during phase 2 of the curriculum, three-year professional skills program whose main objective is to enhance students' clinical skills. Within this program, students are trained in the secondary care hospital setting and the professional skills lab. However, despite those three years of training, students were found to lack clinical skills when they transferred to phase 3 (their clerkship phase). For this reason, the Department of Family & Community Medicine (FAMCO) took the lead in developing an intensive clinical skills training program for a six-week period in various primary health care centers (PHCCSs) to be carried out and overseen by qualified family physicians (FPs). These PHCCS are distributed around the country where all the primary care services are provided free of charge for the population. The services are offered by qualified family physicians that each have their own list of patients registered with them. There are three classes of PHCCS in Bahrain, A, B, and C depending on the population size where in class A most of the medical students' are trained. Each center, beside the family medicine clinics, has laboratory, radiology, ante- and postnatal, dietitian, health education, minor surgery, and emergency services. Students in small groups have the opportunity here to interact and examine real patients and be trained in all of the center's sections.

 The literature has shown that clinical training could occur through engagement and opportunity. Engagement in learning appears to be developed through four essential elements: recognition, respect, relevance, and emotion. Clinical Opportunities include the availability of patient encounters [1]. It is well documented that medical students receive their best training when this is done in PHCCS, due to many factors such as small group sessions [1, 2] and the presence of real patients for hands-on training which includes the development of effective patient interactions along with understanding chronic diseases [2]. It is reported that students in family medicine (FM) conduct physical examination more frequently and gain experience in both the breadth and depth of primary care (PC) [3]. The increased professionalization of teaching in PC results in better training and cost containment and are improved quality of health care at the community level [4].

PHCCS have become a critical element in training medical students, since enhancing interpersonal doctor-patient communication and clinical skills were perceived to be the most positive learning outcomes of these longitudinal ambulatory clerkships [5]. 

This study was done to determine the effectiveness of the newly developed clinical skills module in the PHCCSs in improving students' clinical skills and collecting feedback on their experience.

## 2. Methods 

A new clinical training module was developed for Year 4 medical students at CMMS (total 130) during the academic year 2011-2012. The module consisted of an intensive six-week training program. Preparation for this module lasted for almost one year and was done by a group of department members who examined every aspect of this course. Two training guideline booklets were developed (one for students and the other for tutors). Qualified family physicians were carefully selected, oriented, and recruited to implement the program. Module objectives, the outcomes, plus processes of implementation, and assessment were specified and approved by various committees in the college, including the College Council. Each group consisted of two students assigned to one tutor who spent four hours per session in the PHC clinic. Students were assessed for their level of clinical skills at the onset of the program using two methods; first, by student self-assessment; second, by tutors conducting a clinical exam. For both, a questionnaire was designed using a Likert scale from 1 to 5, assessing areas related to communication skills and examination of vital signs, eye, ear, nose, and throat (EENT), as well as chest, abdomen, and musculoskeletal examinations. Later, after six weeks, students were again asked to complete a similar questionnaire assessing their clinical skills achievement and their clinical skills were also assessed by their tutors. The information gathered from all four stages was entered and analyzed using the Statistical Package of Social Sciences (SPSS, Version 20.0). Data was presented as mean ± SD (standard deviation) and a two-sample *t*-test was used to test the significant difference between skills level at the beginning and end of the program. *P* values less than 0.05 were considered statistically significant.

## 3. Results 

A total of 130 students were enrolled in this module. Student feedback regarding this program was very encouraging. Most thought it one of the best training programs they ever experienced and indicated that it gave them the opportunity to learn many skills they lacked or felt not confident about. Students also indicated that the program period should be extended to longer than six weeks and that they preferred the professional skills training to be conducted in the PHC rather than the hospital setting.

### 3.1. Student Self-Assessment

At the beginning and end of the program students were asked to assess themselves in the following areas:

- communication skills, which included history taking and maintaining patient records;
- examination: vital signs (recording temperature, pulse, blood pressure, respiratory, and heart rates); eye, ear, nose, and throat examination skills; head and neck examination, including lymph nodes; chest examination (heart and lung); abdominal examination; musculoskeletal and joint examination.

Table 1 shows a comparison between students' self-assessment at the start and end of the program, indicating highly significant differences at the beginning and end of the training program. Almost all students felt they improved greatly in their acquisition of various skills. Overall, self-assessment was positive with a mean difference of 0.87 units (*P* value <0.001). History taking was the most improved skill at the end of the program whereas musculoskeletal exam skill was the least improved (*P* value =0.001).

### 3.2. Tutor Assessment

Tutors examined students at the beginning of the program to get an idea of their level of skills. At the end of the program, the same exam was administered to determine what difference the training program made on these students.  Table 2 shows a highly significant difference at the start and end of the training program, both in acquisition of communication and clinical skills. Overall tutor assessment was highly positive, with a mean difference of 1.94 units (*P* value <0.001). Based on tutor assessment, by the end of the program, the vital signs examination was the most improved skill (*P* value <0.001).

### 3.3. Student and Tutor Assessment at the Beginning of the Program

Table 3 shows a comparison between student and tutor assessment at the beginning of the training program, indicating a significant difference between student and tutor evaluations in that students overestimated their clinical skills ability at the start of the program. The most significant difference between both groups was in the area of musculoskeletal exam skills with a mean deference of 1.54 units (*P* value <0.001) (i.e., tutors' assessment was much lower than students' one in this area). The lowest difference was in head and neck skills with a mean difference of 0.55 units (*P* value <0.001).

### 3.4. Student and Tutor Assessment at End of Program

Table 4 shows a comparison between student self-assessment and tutor assessment (using a clinical examination) at the end of the training program. It shows no significant difference between student and tutor evaluation at the end of the training program which indicates an improvement in students' clinical skills. It is interesting to note here that students underevaluated themselves in comparison to their tutors' assessment in this area.

## 4. Discussion 

In the past, medical schools have been challenged to train doctors competently to respond to community health care needs. To this end, many reforms in medical education have been made, refocusing curricula on the need to produce generalist physicians through a problem-based, student-centered, community-oriented, integrated approach to instruction [6]. Hence, community-based education is an important strategy for training students appropriately to deliver primary health care services in the future [7]. 

In 1993, the World Federation for Medical Education Summit in Edinburgh called for bold, clear, attractive, and feasible strategies to equip doctors with the necessary skills for future health care. Skills are also needed for shaping the future of health care services in order to form partnerships with other professionals in communities, to promote PHC and to respond appropriately within the cultural context. Strategies for effective skills training include linking skills to student knowledge and attitude, proper selection of students with aptitude and motivation, training through practice along with feedback, and training teachers and assessors, plus the reinforcement of all these skills after graduation from medical school [8].

One of the main objectives of any medical school is to graduate doctors who are efficient and competent in communication and clinical skills, the core areas of competency for medical students [9]. To accomplish this, various schools have used many different training programs. However, it is vastly important to appropriately train the trainer who will carry that responsibility. The training environment is also critical. Recent studies indicate students may encounter problems when applying clinical skills learned in a skills laboratory to actual patients. To facilitate this transition, it has been recommended that medical schools include patient contact early in the preclinical curriculum [10]. However, few studies show students' early clinical skill development is not influenced by the educational setting [11]. In the end, the choice remains with each college. Some opt to train students in a secondary care setting; others in the professional skills lab, while others prefer primary health care centers. Studies indicate that clerkship students who participate in early clinical experiences in PHCCSs feel better prepared to perform clinical skills during their first clerkships, compared to their peers who had only practiced in a clinical skills laboratory [12]. Whatever the training environment is, there are many factors playing a role in the successful training program. Pearson and Lucas, in 2011, indicated that clinical learning occurs through engagement and opportunity with four elements playing roles in engagement: recognition, respect, relevance, and emotion. Opportunity includes the availability of patient encounters [1]. All these factors, plus the environment, must be appropriately present in PHC and Family Medicine (FM). In FM, training is usually observational and students usually have vast opportunities to learn hands-on patient care. Given a more holistic view of health care and its generalist nature, learning in the primary health care setting is reality based [4]. Various studies have shown that students in family medicine rotation conducted physical examinations more frequently than in any other training activity [3]. Also, the increased number of qualified professional trainers in primary care resulted in better training, cost containment, and improved quality of health care at the community level [4]. In the USA, ambulatory primary care has become a critical element in medical education and enhancements in interpersonal communication and clinical skills were perceived to be two of the most positive learning outcomes [5]. Moreover, the literature has indicated that students develop better clinical skills in the PHC training setting than in the hospital [13].

Students may encounter difficulties in the clerkship phase when they apply clinical skills acquired during their preclinical studies. Therefore, early clinical exposure in the preclinical phase has been recommended to reduce these problems [12]. Such benefits were also observed in our study.

In this study we tried to introduce clinical skills training in PHCCS assuming it to be the optimal place to train medical students. Students are introduced to communication and clinical skills through interactive sessions, using direct contact between students and patients in actual clinical sessions. Students indicated that the number and quality of skills acquired during their short period in PHCCS was equal to all three years' worth of training in the preclerkship phase. Students also responded positively in regards to their experiences in PHCCS, mentioning the broad spectrum of clinical conditions available to be observed and the greater opportunity to acquire clinical skills. Similar findings were reported elsewhere [14]. Students related these benefits to various factors: small groups in their training sessions, the availability of real patients, the opportunity to communicate with and examine patients directly, and the variety of cases, plus the dedication of the tutors. 

Early clinical experiences in PHCCSs impacted positively on students' confidence, clinical reasoning, and interpersonal communication [8]. At Maastricht University, the integration of knowledge, skills, and attitudes in preclinical medical education is promoted by an “Adoption Program”, in which students carry out assignments in a general practice setting. Coaching by a GP guided students in carrying out their assignments [15]. Therefore, general practitioners working as clinical instructors are highly able to influence medical students' level of community-based learning [16]. 

As suggested by the literature, our students' experience shows that direct exposure to real patients plus practice in the community health environment is an effective training approach, broadening students' education by offering them a community perspective on health and disease [17]. 

Although there are several models for teaching how to acquire and conduct physical examinations, few are designed specifically to teach that skill. There is little evidence to select any one model over another. We propose an approach which adopts several key features of each of these models [18]. 

## 5. Conclusion 

Grant and Robling (2006) stated that after Tomorrow's Doctors was published, resulting in increasing numbers of students being admitted to medical schools, it became necessary to involve more general practitioners (family physicians) in undergraduate medical education [14]. Our experience shows that a community-based training module enables our students to achieve more of their important learning objectives such as clinical skills and that qualified FPs probably make the best instructors to equip medical students with these valuable skills.

## Figures and Tables

**Table 1:** Comparison of student self-assessment at the beginning and end of the program.

Skill	Start	End	Mean	*P* value
	Mean ± SD	Mean ± SD	difference	
History taking	3.09 ± 1.14	4.25 ± 0.78	1.16	<0.001
Vital signs examination	3.55 ± 1.16	4.48 ± 0.70	0.93	<0.001
Head and neck examination	2.94 ± 1.13	3.98 ± 0.93	1.04	<0.001
Chest examination	3.13 ± 1.28	4.26 ± 0.81	1.13	<0.001
Musculoskeletal examination	3.77 ± 1.09	4.20 ± 0.92	0.43	0.001
Overall	3.13 ± 0.93	4.00 ± 0.62	0.87	<0.001

**Table 2:** Comparison between tutor assessment at beginning and end of program.

Skill	Start	End	Mean	*P* value
	Mean ± SD	Mean ± SD	difference	
History taking	2.37 ± 0.91	4.29 ± 0.71	1.93	<0.001
Vital signs examination	2.37 ± 1.03	4.56 ± 0.64	2.19	<0.001
Head and neck	2.39 ± 0.91	4.31 ± 0.63	1.92	<0.001
Chest examination	2.28 ± 0.89	4.39 ± 0.71	2.12	<0.001
Abdomen/groin examination	2.38 ± 0.87	4.38 ± 0.71	1.99	<0.001
Musculoskeletal examination	2.23 ± 0.99	4.32 ± 0.74	2.10	<0.001
Neurological examination	2.37 ± 1.10	4.27 ± 0.72	1.90	<0.001
Overall	2.46 ± 0.90	4.40 ± 0.60	1.94	<0.001

**Table 3:** Comparison between student and tutor assessment at the beginning of the program.

Skill	Students	Tutors	Mean	*P* value
	Mean ± SD	Mean ± SD	difference	
History taking	3.09 ± 1.14	2.37 ± 0.91	0.72	<0.001
Vital signs examination	3.55 ± 1.16	2.37 ± 1.03	1.18	<0.001
Head and neck examination	2.94 ± 1.13	2.39 ± 0.91	0.55	<0.001
Chest examination	3.13 ± 1.28	2.28 ± .089	0.85	<0.001
Abdomen/groin examination	3.39 ± 1.16	2.38 ± 0.87	1.01	<0.001
Musculoskeletal examination	3.77 ± 1.09	2.23 ± 0.99	1.54	<0.001
Overall	3.13 ± 0.93	2.46 ± 0.91	0.68	<0.001

**Table 4:** Comparison between student and tutor assessment at the end of program.

Skill	Students	Tutors	Mean	*P* value
	Mean ± SD	Mean ± SD	difference	
History taking	4.25 ± 0.78	4.29 ± 0.71	−0.04	0.647
Vital signs examination	4.48 ± 0.70	4.58 ± 0.63	−0.10	0.226
Head and neck examination	3.98 ± .093	4.33 ± 0.63	−0.36	0.000
Chest examination	4.26 ± 0.81	4.37 ± 0.72	−0.11	0.234
Abdomen/groin examination	4.28 ± 0.90	4.38 ± 0.70	−0.10	0.329
Musculoskeletal examination	4.20 ± 0.92	4.36 ± 0.74	−0.16	0.122
Overall	4.00 ± 0.62	4.39 ± 0.60	−0.38	0.000

## Abbreviations

AGU: Arabian Gulf University
CMMS: College of Medicine and Medical Science
FAMCO: Department of Family & Community Medicine
FM: Family medicine
FP: Family physician
PHC: Primary health care
PHHC: Primary health care center.
